# Comment on: “Double pituitary adenoma associated with acromegaly and hyperprolactinemia: a case report”

**DOI:** 10.20945/2359-4292-2025-0248

**Published:** 2025-10-28

**Authors:** Miriam da Costa de Oliveira, Matheus Nejar Coan, Diego Paixão Côrtes Aguiar

**Affiliations:** 1 Universidade Federal de Ciências da Saúde de Porto Alegre, RS, Brasil

Dear Editor,

Falcone and cols., in a recent article published in *Archives of Endocrinology and Metabolism* (v.69(^2^)), titled *Double pituitary adenoma associated with acromegaly and hyperprolactinemia: a case report*, described a double adenoma in a patient in which surgery led to remission of the disease (^1^). In their conclusions, they emphasized the necessity of careful evaluation of the double lesions to obtain optimal surgical outcomes. This recommendation has also been cited in recently published reviews (^2,3^). The motivation for writing this letter is to share our experience with three of these uncommon cases and to reinforce the therapeutic challenge of missing the second lesion during surgery and the consequent absence of remission, as we observed in a patient with Cushing’s disease.

The MRI diagnosed double noncontiguous pituitary tumors are a rare condition. The confirmatory diagnosis, made by immunohistochemistry (IH) analysis showing adenomatous tissue in the two lesions and immunohistochemistry showing similar or different pituitary hormones or transcription factors, is not always possible since the clinical expression is sometimes of hypersecretion of prolactin that not demands surgery intervention, or, in other cases, there is no clinical repercussion in the presence of microlesions.

Between 2021 and 2025, we followed three of these cases, two male patients and one female, aged 55, 32, and 43 years, respectively. In one case, the patient presented two non-functioning macroadenomas (1.9 x 1.8 x 1.5 and 1.8 x 1.4 x 1.3 cm); in the second one, two microadenomas associated with Cushing’s Disease; and in the third case, microprolactinoma(s). What deserves attention in this small series is the incidental nature of the diagnosis in two of the cases, one of them investigated by psychiatric disturbance and the other by chronic migraine.

In the patient with non-functioning macroadenomas, the tissue showed IH expression of LH. We have not yet obtained the post-surgical imaging of this patient. The patient with Cushing’s disease underwent transsphenoidal surgery with positive tissue IH for ACTH. New imaging was done and showed the persistence of one of the lesions. The patient underwent reintervention, with an IH examination showing PRL+GH+LH, and hypercortisolemia remained. The third patient is currently using cabergoline to treat the hyperprolactinemia.

In conclusion, the unsuccessful experience with one of the three new cases presented serves to reinforce the words of Falcone and cols. that in the case of double lesions, a careful exploration of the sella must be performed to obtain a better surgical outcome.

## Data Availability

datasets related to this article will be available upon request to the corresponding author.

## References

[1] Falcone MGG, Abbati SG, Sosa S, Lima AP, Peralta F, Danilowicz K (2025). Double pituitary adenoma associated with acromegaly and
hyperprolactinemia: a case report. Arch Endocrinol Metab.

[2] Zhang Y, Gong X, Pu J, Liu J, Ye Z, Zhu H (2024). Double pituitary adenomas: report of two cases and systematic
review of the literature. Front Endocrinol (Lausanne).

[3] Reese JC, Zervos TM, Rock J, Tabbarah A, Noushmehr H, Herrgott G (2024). A rare case of double pituitary prolactinomas: the diagnostic
application of intraoperative ultrasonography and DNA methylation
markers. Arch Endocrinol Metab..

